# d-Amino Acids and Classical Neurotransmitters in Healthy and Type 2 Diabetes-Affected Human Pancreatic Islets of Langerhans

**DOI:** 10.3390/metabo12090799

**Published:** 2022-08-27

**Authors:** Cindy J. Lee, Jack H. Schnieders, Stanislav S. Rubakhin, Amit V. Patel, Chengyang Liu, Ali Naji, Jonathan V. Sweedler

**Affiliations:** 1 Department of Chemistry, The Beckman Institute, University of Illinois Urbana-Champaign, Urbana, IL 61801, USA; 2 Department of Surgery, Perelman School of Medicine, University of Pennsylvania, Philadelphia, PA 19104, USA

**Keywords:** d-amino acids, amino acid, neurotransmitter, cell signaling, pancreatic islet, endocrine system, diabetes, chiral analysis, laser-induced fluorescence, mass spectrometry

## Abstract

The pancreatic islets of Langerhans are clusters of cells that function as endocrine units synthesizing and releasing insulin and a range of additional peptide hormones. The structural and chemical characteristics of islets change during type 2 diabetes development. Although a range of metabolites including neurotransmitters has been reported in rodent islets, the involvement of these cell-to-cell signaling molecules within human pancreatic islets in the pathophysiology of type 2 diabetes is not well known, despite studies suggesting that these molecules impact intra- and inter-islet signaling pathways. We characterize the enigmatic cell-to-cell signaling molecules, d-serine (d-Ser) and d-aspartate (d-Asp), along with multiple classical neurotransmitters and related molecules, in healthy versus type 2 diabetes-affected human islets using capillary electrophoresis separations. Significantly reduced d-Ser percentage and gamma-aminobutyric acid (GABA) levels were found in type 2 diabetes-affected islets compared to healthy islets. In addition, the negative correlations of many of the signaling molecules, such as d-Ser percentage (r = −0.35), d-Asp (r = −0.32), serotonin (r = −0.42), and GABA (r = −0.39) levels, with hemoglobin A1c (HbA1c) levels and thus with the progression of type 2 diabetes further demonstrate the disruption in intra- or inter-islet signaling pathways and suggest that these cell-to-cell signaling molecules may be potential therapeutic targets.

## 1. Introduction

Pancreatic islets are mini-organs that are composed of functionally and biochemically heterogeneous cells including endocrine cells that secrete different glucose-regulating hormones [1]. The canonical insulin-producing beta cells comprise 50–60% of endocrine cell populations of human islets and are involved in cell-to-cell signaling leading to glucose storage [2]. In addition, glucagon-producing alpha cells, making up 30–50% of human islets, promote blood glucose elevation during hypoglycemia [3]. Together, cellular composition of these pancreatic islet cells, as well as cell–cell interactions, plays a crucial role in the normal and pathological functioning of organisms including beta cell dysfunction and glucose homeostasis dysregulation during type 2 diabetes [4,5,6,7].

Besides the well-known peptide hormones, pancreatic islets contain a number of cell-to-cell signaling molecules including serotonin [8], GABA [9,10], and acetylcholine [11], which have been detected in human pancreatic islets through immunohistochemical staining or the secretion from islets. These classical neurotransmitters are involved in intra-islet communication and in modulating the release of a range of hormones in islets [12,13]. 

In addition to classical cell-to-cell signaling molecules, recent evidence suggests the possible autocrine or paracrine function of other less common cell–cell signaling molecules—the d-amino acids (d-AAs) [14]. d-AAs, enigmatic endogenous cell-to-cell signaling molecules mostly found in the endocrine and central nervous systems, were denoted in human and rodent pancreatic islets largely through immunohistochemical staining [15,16,17]. Origins of these molecules are traced to microbiota, diet, and for some d-AAs, endogenous synthesis [18]. For example, serine racemase, the enzyme that produces d-Ser from l-serine (l-Ser), is expressed in both rodent and human beta cells [15], while d-Asp is detected in rodent alpha cells [16]. Functionally important, d-Ser [19] and d-Asp [20] bind to the subunits of the *N*-methyl-d-aspartate receptor (NMDAR), which is mainly expressed in islet beta cells [21,22,23]. As NMDAR antagonists, like dextromethorphan, have been shown to increase serum insulin and lower blood glucose in type 2 diabetes patients [24,25], the potential signaling roles of these d-AAs through the NMDARs in human islets in vivo are suggested. 

Considerable progress has been made in elucidating the involvement of cell-to-cell signaling molecules in islet biology. However, the changes in the levels of the aforementioned signaling molecules in normal and pathological functions of human islets are not clear. Here, we compared the levels of d-AAs (i.e., d-Ser and d-Asp) and 11 neurotransmitters and related molecules in isolated healthy, prediabetes, and type 2 diabetes-affected human islets using chiral capillary electrophoresis (CE) separation with laser-induced fluorescence (LIF) detection and microfluidic CE coupled to mass spectrometry (MS). As several molecules correlate with disease, this work reveals a possible relationship between the pathophysiology of type 2 diabetes and the levels of endogenous signaling molecules, which can be further exploited as targets to understand the pancreatic regulation of glucose homeostasis.

## 2. Materials and Methods

### 2.1. Materials and Chemicals

All materials and chemicals were purchased from Sigma-Aldrich (St. Louis, MO, USA) or Fisher Scientific (Hampton, NH, USA) unless stated otherwise. The purity of each reagent was established by the vendors and validated by us using control measurements.

### 2.2. Collection of Human Pancreatic Islets

Human islets for research were provided by the Human Pancreas Procurement and Analysis Program (HPPAP) at the University of Pennsylvania, which is part of the Integrated Islet Distribution Program (IIDP; https://iidp.coh.org/, accessed on 3 August 2022), and along with other centers, formed the Clinical Islet Transplant consortium (CIT). Another source of human islets was the Alberta Diabetes Institute IsletCore (ADI) at the University of Alberta in Edmonton (http://www.bcell.org/adi-isletcore.html, accessed on 3 August 2022) with the assistance of the Human Organ Procurement and Exchange (HOPE) program, Trillium Gift of Life Network (TGLN) and other Canadian organ procurement organizations. Islet isolations were approved by the Institutional Review Board at the University of Pennsylvania (826489) and the Human Research Ethics Board at the University of Alberta (Pro00013094). All live, transplant-quality human pancreatic islets, in addition to prediabtes and diabetes-affected islets, were isolated from the deceased donors following the guidelines of each institute [26,27] to facilitate human islet research to understand the pathophysiology of diabetes. All donors’ families gave informed consent for the use of pancreatic tissue in research. Body mass index (BMI), HbA1c levels, and health status of many (but not all) of the donors were provided by the HPPAP and ADI. The health status was determined based on a donor’s previous medical history (if available) or clinical diagnosis using HbA1c levels by each institute. 

### 2.3. Human Pancreatic Islet Processing

Anonymized samples were shipped overnight and processed upon arrival following the project approved by the Institutional Biosafety Committee (IBC, University of Illinois Urbana-Champaign). Specifically, the pancreatic islet equivalents (IEQs) were divided into aliquots based on the calculated numbers of IEQs provided by the senders. Resulting samples were washed with cold (4 °C) modified Gey’s balanced salt solution (mGBSS) containing the following: 1.5 mM CaCl_2_, 4.9 mM KCl, 0.2 mM KH_2_PO_4_, 11 mM MgCl_2_, 0.3 mM MgSO_4_, 138 mM NaCl, 27.7 mM NaHCO_3_, 0.8 mM Na_2_HPO_4_, and 25 mM HEPES, pH 7.2. Washed aliquots were transferred into tubes containing 600 µL of methanol for analyte extraction. The resulting samples were stored at −80 °C until analysis. 

### 2.4. Amino Acid Extraction

Removed from a −80 °C environment, samples were kept on ice during the analyte extraction procedure. Water was added to all samples to make 80:20 (v:v) methanol:water analyte extraction media. Analytes were extracted from samples by vortexing and sonicating for 10 min. The samples were then centrifuged at 10,000× *g* for 5 min at 4 °C. The supernatant was dried in a SpeedVac (Eppendorf Vacufuge plus). While drying, 1000 μL of water was added to the pellet for an additional round of analyte extraction. The supernatant collected after sample centrifugation was combined with the previously dried analyte extracts. Combined samples were dried again in a Speedvac and reconstituted in 30 μL of LC-MS grade water. The Micro BCA Protein Assay kit or the Pierce BCA Protein Assay Kit (ThermoFisher, Waltham, MA, USA) was utilized to determine the total protein amounts from aliquots of the extracted islet samples according to the manufacturer’s instructions. The remaining samples were stored at −80 °C until further analysis.

### 2.5. Capillary Zone Electrophoresis (CZE)-LIF for Chiral Separation

#### 2.5.1. Amino Acid Derivatization

Aqueous solutions prepared in either LC-MS grade water or ultrapure water (Milli-Q Direct Water Purification System, MilliporeSigma, Burlington, MA, USA) were used for CE-LIF measurement. For CE-LIF detection, d/l-Ser and d/l-Asp were derivatized by reaction with naphthalene-2,3-dicarboxaldehyde (NDA) (Invitrogen, Carlsbad, CA, USA). A 4 μL mixture of an aliquot of the islet extract, 20 mM potassium cyanide (KCN) in 100 mM borate buffer, and 20 mM NDA in acetonitrile (ACN) was prepared in a 1:2:1 volume ratio. The mixture was allowed to react for 2 min in the dark at room temperature and then diluted to 10 μL of total volume by water. The samples were further desalted using a procedure similar to one previously used by our group [28] (see the Supplementary Materials). For quantitation using linear calibration curves, d/l-Ser or d/l-Asp standards of different concentrations ranging from 0.025–100 μM for d and 0.125–500 μM for l were prepared, NDA-derivatized as previously described, and diluted to 100 μL by water for CE-LIF analysis. 

#### 2.5.2. Enzyme Treatment for Confirmation of d-AA Identification 

When the detection of endogenous d-AAs relies on standard migration time matching, it is recommended that the peak identity assignment is confirmed via the enzymatic degradation of the targeted compound and the disappearance of the corresponding signal [29]. Islet samples were treated with d-amino acid oxidase (DAAO) from porcine kidney (Catalog# A5222, Sigma-Aldrich) and d-aspartate oxidase (dAspO), cloned and purified within our group [28], for confirmation of d-Ser and d-Asp signal identifications, respectively. For d-Ser measurement, an aliquot of sample was mixed with 15 U/mL purified DAAO, 68 μg/mL purified catalase from bovine liver, 5 mM flavin adenine dinucleotide (FAD), and PBS 1X (Gibco, ThermoFisher, Waltham, MA, USA) in a volume ratio of 1:2:1:1:5 (see the Supplementary Materials for enzyme purification). For d-Asp confirmation, 5.4 mg/mL dAspO substituted DAAO in the mixture for the volume ratio of 1:0.6:1:1:6.4. The reaction mixtures were incubated at 37 °C for 24 h in a Bio-Rad T100 thermal cycler. After the reaction, 50 μL of methanol was added to the mixtures, and they were dried in a SpeedVac and stored at −80 °C until analysis. The enzyme-treated samples were reconstituted in water, NDA-derivatized, desalted, and analyzed by CE-LIF.

#### 2.5.3. CZE-LIF

Chiral separations were performed using a PA 800 Plus Pharmaceutical Analysis System equipped with LIF detection (AB SCIEX, Framingham, MA, USA). The system was coupled with a fiber optic cable (OZ Optics, Ottawa, ON, Canada) connected to an external diode laser (56ICS426, Melles Griot, Carlsbad, CA, USA). The 3 mW laser with a centroid wavelength of 440 ± 8 nm was used in the measurements. A band-pass filter of 490 ± 15 nm (Omega Optical, Brattleboro, VT, USA) was selected for detecting the appropriate fluorescence emission band. Bare fused-silica capillaries (Polymicro Technologies, Phoenix, AZ, USA) were used in all separations. The capillaries had the total/effective lengths of 40/30 cm with inner/outer diameters of 50/360 μm. All capillaries were rinsed with 1 M NaOH and water for 1 h each before initial use.

The stock solutions for d-Ser and d-Asp separations were prepared as follows. A 300 mM 2-(*N*-morpholino)ethanesulfonic acid (MES) (pH 6) was prepared in water and pH adjusted with 5 M NaOH. Additionally, a 200 mM potassium bromide (KBr) in water, a 10% (w/v) quaternary ammonium β-cyclodextrin (QAβCD) (CTD Holdings, Alachua, FL, USA) solution in water, and a stock of 1 M citric acid in water were prepared. All mentioned stock solutions were stored at 4 °C.

For the d-Ser separation, the separation buffer with final concentrations of 62 mM MES (pH 6), 7 mM KBr, and 330 ppm QAβCD was prepared daily in water and adjusted to pH 6.8. The d-Asp separation buffer (pH 4.7) was also prepared daily in water with final concentrations of 30 mM citric acid, 20 mM KBr, 133 ppm QAβCD, and 50 mM NaOH. d-Ser and d-Asp separations were performed using reverse polarity at 10 kV and cartridge temperature at 20 °C. Samples were injected hydrodynamically with a pressure of 0.5 psi for 5.0 s. Between runs, the CE capillary was pressure-rinsed (20.0 psi) with 1 M NaOH (2 min), water (4 min), and separation buffer (2 min). The OriginPro 2022 (9.9) software (Origin Lab Corp., Northampton, MA, USA) was used for data processing and analysis.

### 2.6. ZipChip-CZE-ESI-MS for Neurotransmitters

The ZipChip CE ion source (908 Devices, Boston, MA, USA) was installed in front of the inlet of a Bruker maXis 4G Quadruple Time-of-Flight Mass Spectrometer (Bruker Corp., Billerica, MA, USA). The HS ZipChips (part no. 810-00195) and ZipChip Metabolites Assay Kit (part no. 850-00033) (908 Devices) were used for metabolite separation analysis following the vendor’s recommended parameters. The separation was performed using the HS Metabolites Default method from the vendor using the following ZipChip parameters: field strength start, 1000 V/cm; background electrolyte (BGE) type, metabolites; injection volume, 5 nL; pressure assist, enabled; pressure assist start time, 2.0 min; replicate delay, 20 s; analysis time, 3.0 min. BGE refresh was performed every 6 runs. Only cation separation was performed on the ZipChip. Acquisition parameters for MS analysis were as recommended by the vendor: source, CaptiveSpray; full scan mass to charge ratio (*m/z*) range, 70–1000; spectra rate, 5.00 Hz; nanoBooster, 2.3 psi; dry gas, 1.0 L/min; dry temp, 200 °C; ion polarity, positive. The mass spectrometer was regularly calibrated in the *m/z* range of 50–500 using a direct infusion of 15 mM sodium formate in a 1:1 ratio of ACN and water via the ESI source. 

For Zipchip-CE-ESI-MS analysis, the dried samples after desalting (see the Supplementary Materials) were reconstituted in acidified Metabolites Diluent (908 Devices) containing 1 μM quinine as an internal control. Stock standards ranging from 0.1–150 μM for GABA, 0.1–10 μM for glutamate, synephrine, norepinephrine, and l-DOPA, and 0.1–5 μM for serotonin, acetylcholine, dopamine, tyramine, epinephrine, and tryptamine were prepared in water with 1% formic acid. The standards were diluted 10-fold in the acidified Metabolites Diluent to construct linear calibration curves for quantitation. A 5 μL aliquot of sample/standard was manually loaded onto the ZipChip system for three replicate measurements. Data were processed using the Bruker DataAnalysis (Bruker Corp.).

### 2.7. Statistics

The sample size for each healthy, prediabetes, and type 2 diabetes-affected individual was *n* = 9, *n* = 4, and *n* = 9, respectively. For comparison of analyte levels or ratios in healthy versus type 2 diabetes samples, the Grubb’s outlier test was performed using GraphPad to identify outliers, and the outliers were then excluded from the dataset for the two-tailed Student’s t-test. Pearson correlation tests were used to assess the correlation of each analyte level or ratio to BMI and HbA1c. Statistical analyses were performed using OriginPro (OriginLab Corp.). Significant differences were stated at *p* < 0.05.

## 3. Results

### 3.1. Determination of d-AAs in Islets of Healthy and Type 2 Diabetes-Diagnosed Individuals

**Figure 1:**
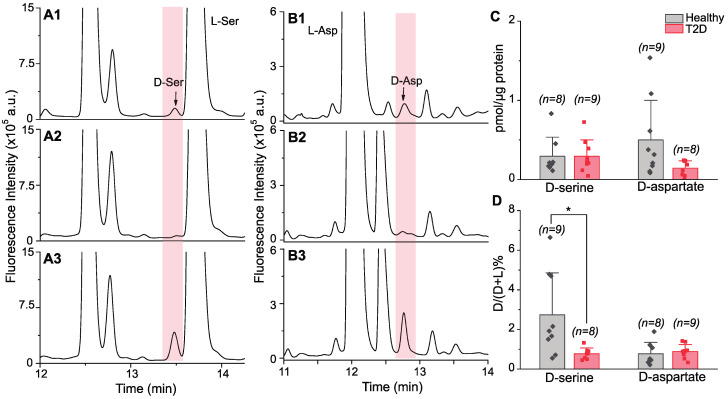
Representative CZE-LIF electropherograms and the results of quantitative analysis of d-Ser and d-Asp in healthy versus type 2 diabetes (T2D)-affected human pancreatic islets. Electropherograms acquired for samples (**A1**) not treated with DAAO enzyme, (**A2**) treated with DAAO enzyme, and (**A3**) treated with DAAO and spiked with d-Ser standard. Electropherograms acquired for samples (**B1**) not treated with dAspO enzyme, (**B2**) treated with dAspO enzyme, and (**B3**) treated with dAspO and spiked with d-Asp standard. (**C**) d-AA levels and (**D**) d-AA percentages. %d = d/(d + l) × 100. Values represent mean ± SD of 8–9 human islet samples after the removal of outliers. * *p* < 0.05.

A number of studies demonstrated the presence of serine racemase [15] and d-Asp [16] in rodent and human islets by immunohistochemical staining. In the present investigation, we measured d-Ser and d-Asp levels in human islets using chiral CE-LIF (Figure 1A,B), for its high sensitivity and low sample amount requirement [30], which is ideal for limited human samples. However, analyte migration time matching and standard spiking are not sufficient for high-confidence peak identification. Therefore, an additional step of enzymatic analyte degradation was used to increase the confidence. DAAO and dAspO mediate oxidative digestion of non-acidic d-AAs and acidic d-AAs, respectively [18,31]. As expected, the signals for d-Ser and d-Asp disappeared in electropherograms collected after the enzymatic treatments (Figure 1A2,B2).

Figure 1C,D shows the amounts of d-AAs found in islets of 9 healthy and 9 type 2 diabetes-affected humans, in addition to d-AA percentages, which are calculated by the level of d-AA to the total level of both corresponding AA enantiomers (i.e., %d = d/(d + l) × 100). In Figure 1C, the average amounts of d-Ser were 0.29 ± 0.24 pmole/μg protein and 0.29 ± 0.21 pmole/μg protein in healthy and type 2 diabetes-affected human islets, respectively, demonstrating similar levels of d-Ser between the two groups. In the case of d-Asp, although no significant difference in the levels of d-Asp was found between the two groups, there was a noticeable tendency for its lower levels in type 2 diabetes-affected islets (0.50 ± 0.50 pmole/μg protein in healthy and 0.14 ± 0.09 pmole/μg protein in type 2 diabetes-affected islets). On the other hand, when comparing d-AA percentages in islets of healthy and type 2 diabetes-diagnosed individuals as shown in Figure 1D, the d-Ser percentage significantly decreased from 2.7 ± 2.1% in healthy islets to 0.8 ± 0.3% in type 2 diabetes-affected islets, while d-Asp percentages were similar between the two groups (0.8 ± 0.6% in healthy and 0.9 ± 0.4% in type 2 diabetes-affected islets). Our data demonstrated that the significant difference in d-Ser percentage was due to higher l-Ser levels in type 2 diabetes-affected islets (Supplementary Figure S1).

### 3.2. Determination of Neurotransmitters in Islets of Healthy and Type 2 Diabetes-Diagnosed Individuals

**Figure 2:**
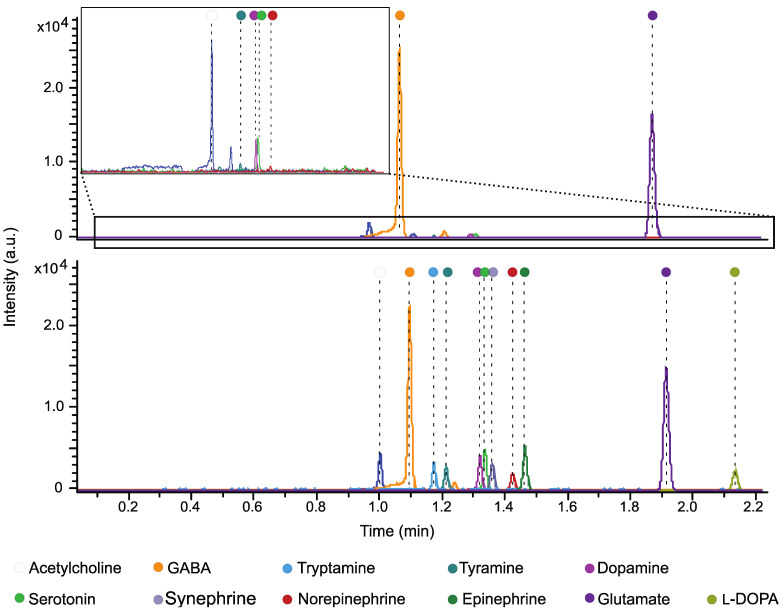
Representative extracted electropherogram of ZipChp CE-MS showing the detection of classical neurotransmitters and related molecules in human islets. (**Top**) sample; (**Bottom**) sample spiked with standards. Inlet in top panel depicts magnified extracted ion electropherograms for five analytes with relatively low signal areas.

To enhance the knowledge of cell-to-cell signaling molecule levels in healthy and type 2 diabetes-affected islets, in addition to d-AAs, we examined 11 endogenous neurotransmitters and related molecules. Because chiral separation was not required for the analysis of targeted molecules including neurotransmitters, we used microfluidic CZE-MS (ZipChip CE-MS) for its fast separation, within 3 min, and higher confidence analyte identification utilizing both migration times and accurate *m/z* values. Figure 2 shows the representative extracted electropherograms of acetylcholine, GABA, tryptamine, tyramine, dopamine, serotonin, synephrine, norepinephrine, epinephrine, glutamate, and l-DOPA detected in a human islet sample and a sample spiked with standards, demonstrating our ability to detect all targeted molecules in the same biological matrix of human islets. 

**Figure 3:**
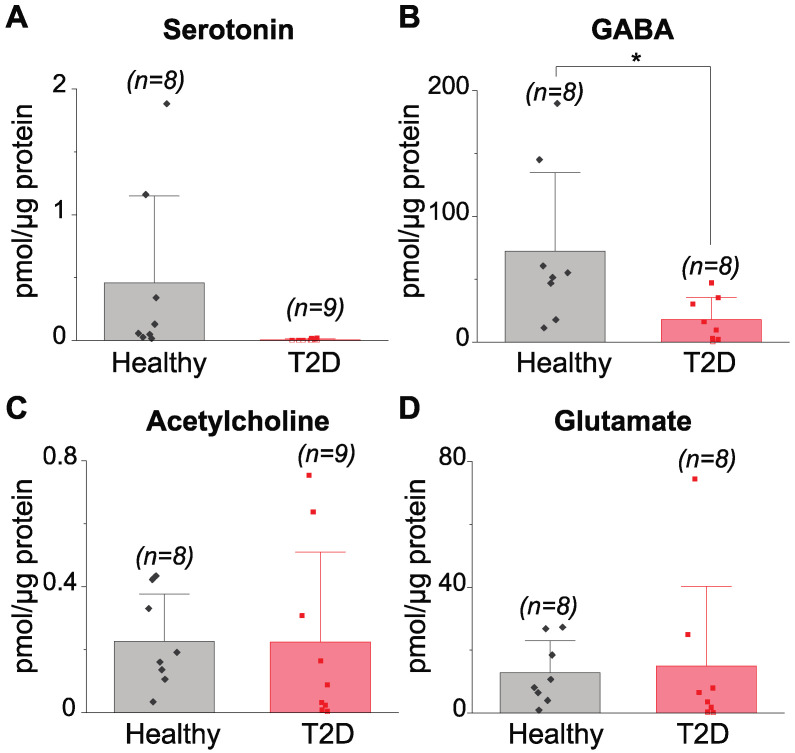
Levels of classical neurotransmitters in healthy versus type 2 diabetes (T2D)-affected human islets. (**A**) serotonin, (**B**) GABA, (**C**) acetylcholine, and (**D**) glutamate. Values represent mean ± SD of 8–9 human islet samples after the removal of outliers. * *p* < 0.05.

A comparison of classical neurotransmitter levels in healthy and type 2 diabetes-affected human islets is shown in Figure 3, and the related data for each analyte measured are summarized in Supplementary Table S1. Serotonin levels in type 2 diabetes-affected islets were either below the limit of detection or very low, if detected, compared to healthy islets. However, due to the large variation within both groups and a relatively small number of samples analyzed, no statistical difference was found between healthy and type 2 diabetes-affected islets (0.46 ± 0.70 pmole/μg protein and 0.0049 ± 0.0076 pmole/μg protein, respectively). On the other hand, a significantly lower GABA level was found in type 2 diabetes-affected islets compared to healthy islets. Acetylcholine and glutamate were detected in all human samples we analyzed at similar levels in both groups. The presence of some other neurotransmitters, such as dopamine, varied from individual to individual and was not different between the examined groups, as shown in Supplementary Table S1. Tryptamine and l-DOPA were not detected in any human islet samples. 

### 3.3. Assessing the Correlation between Analyte Levels or Ratios and Donor Characteristics

**Figure 4:**
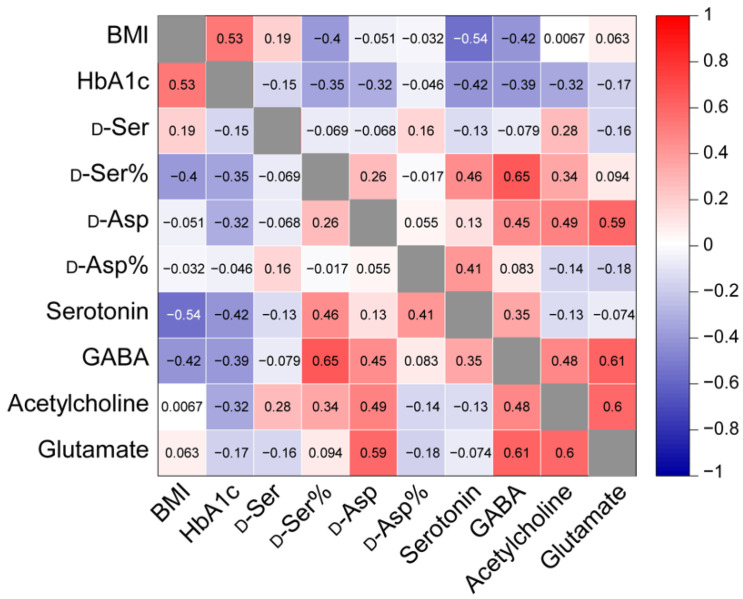
Correlation matrix plot showing Pearson correlation coefficients for selected measured biochemical parameters, BMI, and HbA1c levels. Pearson correlation coefficient of 1 indicates a perfect positive or negative linear relationship between variables, while 0 corresponds to absence of linear relationship.

Several biochemical and phenotypic characteristics, including BMI and HbA1c levels, are associated with type 2 diabetes development and progression [32,33]. We performed a Pearson correlation analysis in Figure 4 to determine possible relations between measured biochemical parameters, including d-AAs and neurotransmitters, and the provided BMI and HbA1c levels. Analytes with levels that varied by individuals rather than the health state were not included in the correlation test. BMI is used as a screening parameter for determining obesity (underweight, <18.5; normal weight, 18.5 to <25; overweight, 25.0 to <30; obese, 30.0 or higher) [32], while HbA1c represents average blood glucose level over 2–3 months and is used for type 2 diabetes diagnosis (healthy <5.7%) [33]. Our work demonstrated a positive correlation between BMI and HbA1c levels (Pearson correlation coefficient (r) = 0.53), which agrees with the report by Boye et al. [34]. Out of the total of 22 donors, 5 healthy, 4 prediabetes, and 7 type 2 diabetes-affected individuals had BMI information available (Supplementary Table S1). The analytes present in the islets were correlated to the BMI of corresponding donors (Supplementary Figure S2). d-Ser percentage (r = −0.40), serotonin (r = −0.54), and GABA (r = −0.42) levels were all inversely correlated to BMI, and thus to obesity. Using 3 healthy, 4 prediabetes, and 6 type 2 diabetes-affected islet samples in Supplementary Table S1, the relationships between the selected analyte and HbA1c levels were also explored (Figure 4 and Supplementary Figure S3). HbA1c was negatively correlated to d-Ser percentage (r = −0.35), d-Asp (r = −0.32), serotonin (r = −0.42), GABA (r = −0.39), and acetylcholine (r = −0.32) levels. Although some of these analytes were not significantly different when comparing data on healthy and type 2 diabetes-affected islets (Figure 1C,D and Figure 3), they showed correlations to BMI and/or HbA1c levels. Several measured analytes also displayed positive or negative correlations amongst each other (Figure 4).

## 4. Discussion

Until now, the presence of d-AAs in pancreatic islets was reported and quantified in rodent islets [35,36]. In the present study, we measured the endogenous d-Ser and d-Asp in human islets for the first time and also compared their levels in healthy versus type 2 diabetes-affected human islets. Our detection of d-Ser is not surprising given previous reports on serine racemase expression in primary human beta cells [15] and human beta-cell lines [37]. Although studies have shown the mixed effects of d-Ser on the rodent islet glucose-stimulated insulin secretion (GSIS) based on the d-Ser concentrations [38,39], as well as opposing outcomes associated with serine racemase expression on the islet GSIS [15,37], we observed similar d-Ser amounts in healthy and type 2 diabetes-affected islets (Figure 1C). This suggests that d-Ser levels in human islets are not affected by beta cell dysfunction in type 2 diabetes. In the case of l-Ser, we measured slight l-Ser elevation in type 2 diabetes-affected islets (Supplementary Figure S1), although both amounts of d-Ser and l-Ser in our study were not statistically different between healthy and type 2 diabetes-affected islets. As l-Ser supplementation is suggested to enhance insulin secretion [40], our observed increase in l-Ser levels may be the result of diseased islets countering the changes in insulin secretion during type 2 diabetes development. This hypothesis may have additional support if higher statistical power is achieved in a study with larger sample sizes. Another possibility is that l-Ser accumulates in type 2 diabetes-affected islets, thus increasing the formation of deoxysphingolipids which causes apoptosis in beta cells to induce type 2 diabetes [40,41]. This latter hypothesis aligns with the l-Ser deficiency and deoxysphingolipids increase in blood/plasma of type 2 diabetes patients [40,42]. Nevertheless, the biochemical mechanisms responsible for d/l-AA profiles in human islets remain unclear and warrant further study. d-Ser percentage, on the other hand, was significantly reduced in type 2 diabetes-affected islets (Figure 1D). Therefore, the d-Ser percentage may be a more consistent biomarker for assisting the diagnosis and determination of type 2 diabetes progression, especially since the d-Ser percentage was negatively correlated to both BMI and HbA1c levels with stronger relationships than either d-Ser or l-Ser levels (Figure 4). As BMI and HbA1c levels were positively correlated (Figure 4), reflecting the higher prevalence of type 2 diabetes in those who are affected by obesity [43], it is not surprising that several signaling molecules are similarly correlated to both BMI and HbA1c levels. 

Although the islet pathology in type 2 diabetes is typically focused on insulin and beta cells, increased glucagon secretion and alpha cell function can also contribute to hyperglycemia in type 2 diabetes [44,45]. The levels of d-Asp, which was detected in glucagon-secreting rodent alpha cells [16], were consistently lower in type 2 diabetes-affected islets in contrast to healthy islets (Figure 1C). Interestingly, the d-Asp level had a negative correlation to the HbA1c level, a characteristic of type 2 diabetes progression (Figure 4). The reduced d-Asp level in type 2 diabetes-affected islets may be due to its enhanced secretion or lower accumulation in the islets during type 2 diabetes progression. The released d-Asp then may have a paracrine role on the neighboring beta cells by binding to NMDAR, where d-Asp acts as an agonist [20], thus causing persistent activation of NMDAR and inhibiting insulin secretion [1]. Our finding of a reduced trend in l-Asp levels in type 2 diabetes-affected islets (Supplementary Figure S1) also aligns with the association of aspartate with increased insulin resistance and reduced insulin secretion [46]. 

Although known as a classical neurotransmitter in the central nervous system, 90% of the serotonin in the body is derived from the enterochromaffin cells located in the gastrointestinal tract [47]. Somewhat surprisingly, serotonin is found in human beta cells and inhibits glucagon release upon increasing glucose levels, while its conflicting effects on insulin release exist [8,47,48,49]. In Figure 3, our direct measurements uncovered a consistently lower level of serotonin in type 2 diabetes-affected islets compared to healthy islets, although limited by statistical power due to the availability of deceased human donors with different health statuses. Nevertheless, together with our use of more than minimum sample size necessary for statistical evaluation and with fewer serotonin receptors found in alpha cells of type 2 diabetes patients via immunostaining [8], our result supports the notion that alpha cells may have a weaker response to the change in glucose level with the lack of serotonin stimulation, leading to the variable release of glucagon as seen in type 2 diabetes. In addition to the negative correlation to HbA1c levels, serotonin levels were negatively related to BMI (Figure 4). Although serotonin-positive cell counts have shown a positive correlation to BMI [8], cell counts do not necessarily correspond to serotonin levels and may indicate a compensatory mechanism to the change in biological systems. Abnormal regulations of serotonin in the whole blood [50] and the brain [51] have been also linked to obesity. It is interesting to speculate whether the serotonin changes observed are due to changes in the gut enterochromaffin cell serotonin levels, providing a distinct molecular link between the gastrointestinal tract and islets.

Higher levels of GABA were found in our study compared to other measured analytes (Supplementary Table S1), in addition to a significant difference in GABA levels between healthy and type 2 diabetes-affected islets (Figure 3). This result agrees with a similar study by Menegaz et al. [9] where lower levels of intracellular GABA and GABA release were observed from islets of type 2 diabetes-exhibiting individuals. Since no change in the GABA synthesizing enzyme (GAD65) was found in healthy versus type 2 diabetes-affected human islets via immunostaining [9], several other factors may influence this parameter including lower GABA synthesis as mentioned by Menegaz et al. through the increased presence of either the inactive form of GAD65 or its inhibitor and more GABA metabolism over GAD65 activation. Nevertheless, with mixed results of intra-islet GABA secretion on insulin release [9,52], the effect of impaired GABA signaling in the diseased islets remains poorly understood. 

Levels of other classical neurotransmitters we measured such as acetylcholine and glutamate were not different between healthy and type 2 diabetes-affected islets (Figure 3). Experimental evidence demonstrates the involvement of glutamate receptors in type 2 diabetes development and beta cell death [1]. This suggests that glutamate formation in islets, even those derived from the TCA cycle in beta cells [1], may result in stable homeostatic glutamate levels regardless of disease state and that the reported overactivation of glutamate receptors may be linked to higher glutamate synthesis and release. Additionally, external sources like plasma-derived glutamate may be responsible for this phenomenon [53]. 

Several measured analytes exhibited different levels of correlation between each other (Figure 4). For example, glutamate and GABA levels had a strong positive relationship, perhaps reflecting glutamate as a precursor for GABA synthesis [1]. The mechanisms of many of the observed relationships in islets warrant further studies. However, the current findings suggest the potential signaling function of d-AAs and neurotransmitters in human islets, making them potential targets for future pharmacological intervention.

## 5. Conclusions

While the autocrine or paracrine role of d-AAs and neurotransmitters has been proposed, prior direct measurement of their levels in human pancreatic islets is limited, especially in type 2 diabetes-affected islets. Here, we uncovered significant reductions in d-Ser percentage and GABA levels, as well as the changes in d-Asp and serotonin levels, in type 2 diabetes-affected human islets compared to healthy islets. The negative correlations of these analyte levels or ratios to type 2 diabetes-associated factors such as HbA1c levels and BMI were further noted. Many aspects of how these signaling molecules are regulated in islet normal and pathological conditions remain unclear. Nevertheless, given the cell–cell signaling roles of these molecules, the observed differences suggest a misregulation of intra-islet chemical communication and provide further pathways to explore for diabetes therapeutics.

## Data Availability

All processed data are presented in the main text and Supplementary Materials. All original data are available and can be shared upon request by contacting the corresponding author.

## Supplementary Materials

The following supporting information can be downloaded at: https://www.mdpi.com/article/10.3390/metabo12090799/s1, Supplementary Materials and Methods; Table S1: Summary of biochemical characteristics of measured human pancreatic islets that varied by individual to individual in three groups: healthy, prediabetes, and type 2 diabetes (T2D)-affected; Figure S1: Levels of l-Ser and l-Asp in healthy versus type 2 diabetes (T2D)-affected human pancreatic islets; Figure S2: Scatter plots of BMI and each d-Ser, d-Asp, and classical neurotransmitters; Figure S3: Scatter plots of HbA1c and each d-Ser, d-Asp, and classical neurotransmitters.
