# Supplementary material for: d-Amino Acids and Classical Neurotransmitters in Healthy and Type 2 Diabetes-Affected Human Pancreatic Islets of Langerhans
Source: Metabolites. 2022 Aug 27;12(9):799. doi: 10.3390/metabo12090799 (PMC9501506; doi:10.3390/metabo12090799)
Supplement: Supplementary file 1 [file metabolites-12-00799-s001.zip › metabolites-1875481-supplementary.pdf]

Supplementary Materials

D-Amino acids and classical neurotransmitters in healthy and type 2 diabetes affected human pancreatic islets of Langerhans

Cindy J. Lee[1], Jack H. Schnieders[1], Stanislav S. Rubakhin[1], Amit V. Patel[1], Chengyang Liu[2], Ali Naji[2], Jonathan V. Sweedler[1,*]

[1] Department of Chemistry and the Beckman Institute, University of Illinois Urbana-Champaign, Urbana, IL 61801, United States

[2] Department of Surgery, Perelman School of medicine, University of Pennsylvania, Philadelphia, PA 19104 United States

[*]Corresponding author: jsweedler@illinois.edu (Jonathan V. Sweedler)

Table of Contents

**Supplementary Materials and Methods**		S-2
**Table S1**	Summary of biochemical characteristics of measured human pancreatic islets that varied by individual to individual in three groups: healthy, prediabetes, and type 2 diabetes (T2D)-affected.	S-3
**Figure S1**	Levels of L-Ser and L-Asp in healthy versus type 2 diabetes (T2D)-affected human pancreatic islets.	S-5
**Figure S2**	Scatter plots of BMI and each D-Ser, D-Asp, and classical neurotransmitters.	S-6
**Figure S3**	Scatter plots of HbA1c and each D-Ser, D-Asp, and classical neurotransmitters.	S-7

Supplementary Materials and Methods:

Capillary zone electrophoresis (CZE)-LIF for chiral separation

Sample desalting

Homemade centrifuge extraction tips were prepared using a 200 µL pipette tip packed with three layers of punched-out Empore SPE Disks (0.7-0.8 mg of polystyrene-divinylbenzene (SDB-XC)). The disk's SDB-XC material was conditioned with 15 µL of ACN and then 45 µL of water. The NDA-derivatized samples were dried in a SpeedVac, reconstituted in a minimum volume of water (typically 5 µL), and loaded onto the tips. The tips were washed with 5 µL of water aliquots twice. The analytes were eluted with 45 µL of ACN. The resulting samples were dried in a SpeedVac and reconstituted in water for CE-LIF analysis.

Enzyme purification for confirmation of D-AA identification

Before the enzymatic treatment, 15 U/mL DAAO and 68 µg/mL catalase, each in PBS 1X (Gibco, ThermoFisher), were prepared using the DAAO from porcine kidney (Catalog# A5222, Sigma-Aldrich) and catalase from bovine liver (Catalog# C30, Sigma-Aldrich). Both reagents underwent further purification, as the presence of interfering compound within the enzyme solution co-eluted with the analyte peak and prevented the confirmation by CE-LIF. 150 µL of each prepared reagent was transferred to 3kDa MWCO filters (Amicon Ultra 0.5 mL centrifugal filters), conditioned with 500 µL of PBS 1X. The reagents were centrifuged at 14,000 × g for 15 min. 150 µL of PBS 1X was added to the same filter and centrifuged again at 14,000 × g for 15 min. The concentrated enzymes were recovered by centrifuging the filters upside down in tubes at 1,000 × g for 2 min. The concentration of each purified enzyme was determined using a NanoDrop and further diluted in PBS 1X to make purified 15 U/mL DAAO and 68 µg/mL catalase.

ZipChip-CZE-ESI-MS for neurotransmitters

Sample desalting

Aliquots of the extracted islet samples stored at -80 °C were dried in a SpeedVac. The resulting samples were resuspended in a 50 µL mixture of isopropanol and ACN in a 4:1 volume ratio and centrifuged at 15,000 × g for 5 min at 4 °C, solubilizing analytes and further cleaning up the samples. The supernatant was dried in a SpeedVac and stored at -80 °C until analysis.

Table S1 Summary of biochemical characteristics of measured human pancreatic islets that varied by individual to individual in three groups: healthy, prediabetes, and type 2 diabetes (T2D)-affected.

Type	Subject No.	BMI	HbA1c (%)	D-Ser	D-Ser (%)	D-Asp	D-Asp (%)	Serotonin	GABA	Ach	Glutamate
**Healthy** (n = 9)	1	n/a	n/a	1.6E-01	4.8	1.7E-01	1.1	2.5E-02	1.1E+01	3.4E-02	1.0E+00
	2	n/a	n/a	8.3E-01	4.7	1.0E-01	0.5	4.7E-02	4.7E+01	1.6E-01	2.7E+01
	3	n/a	n/a	5.8E+00	6.6	1.5E+00	0.5	1.2E+00	7.2E+02	4.3E-01	7.7E+01
	4	n/a	n/a	4.5E-01	0.7	1.1E+00	0.5	1.9E+00	5.5E+01	1.9E-01	2.7E+01
	5	20.3	4.8	1.7E-01	1.5	2.1E-01	3.2*	5.7E-02	1.5E+02	3.3E-01	8.2E+00
	6	26.2	5.3	2.2E-01	2.2	3.2E-01	1.9	3.4E-01	1.9E+02	1.6E+00*	1.9E+01
	7	35	n/a	1.9E-01	1.7	8.2E-02	0.2	1.6E-02	5.2E+01	1.4E-01	4.1E+00
	8	28	n/a	2.1E-01	1.9	3.8E-01	0.4	3.8E+00*	6.1E+01	4.2E-01	6.5E+00
	9	30	5.4	1.1E-01	0.6	6.1E-01	1.2	1.3E-01	1.8E+01	1.1E-01	1.1E+01
**Prediabetes** (n = 4)	10	33.2	6.1	2.6E-01	0.6	1.2E-01	0.8	1.7E-02	1.1E+02	1.6E-01	3.4E+01
	11	27	5.9	2.1E-01	0.5	4.1E-02	0.5	1.4E-01	1.4E+01	6.3E-02	7.4E+00
	12	24	5.8	2.5E-01	0.9	8.0E-02	0.5	1.9E-01	2.1E+01	1.2E-01	6.9E+00
	13	27.5	5.7	1.6E-01	1.1	9.0E-02	1.3	1.1E-01	1.0E+01	5.7E-01	1.5E+01
**T2D** (n = 9)	14	n/a	n/a	2.1E-01	2.9	2.4E-01	0.7	1.8E-02	3.5E+01	3.7E-03	8.0E+00
	15	n/a	6.7	7.3E-01*	1.3	6.5E-01*	0.3	n.d.	1.6E+02	7.5E-01	7.4E+01
	16	38	6.3	1.2E-01	0.4	2.3E-01	0.9	n.d.	3.0E+01	3.1E-01	2.5E+01
	17	35.9	9.7	4.5E-02	0.8	2.0E-02	0.5	1.1E-02	9.5E+00	2.3E-02	1.9E+00
	18	35.8	n/a	3.9E-01	0.9	2.4E-01	1.4	n.d.	2.1E+00	8.7E-02	1.7E-01
	19	37.4	6.4	4.7E-01	0.6	1.9E-01	1.0	n.d.	1.6E+01	6.4E-01	6.6E+00
	20	26.7	6.0	2.0E-01	0.8	1.1E-01	0.9	n.d.	4.7E+01	1.6E-01	2.8E+02*
	21	28	n/a	2.5E-01	1.0	4.6E-02	0.9	n.d.	1.8E-01	8.4E-03	3.7E-01
	22	33	10.8	2.1E-01	0.5	6.2E-02	1.4	1.5E-02	3.1E+00	3.1E-02	3.5E+00

Table S1 (cont.)

Type	Subject No.	Dopamine	Tyramine	Synephrine	Norepinephrine	Epinephrine
**Healthy** (n = 9)	1	9.4E-03	1.9E-02	2.6E-02	n.d.	n.d.
	2	3.7E-02	n.d.	1.3E-01	n.d.	n.d.
	3	n.d.	n.d.	5.0E-01	n.d.	n.d.
	4	n.d.	8.4E-02	4.0E+00	n.d.	n.d.
	5	4.9E-01	n.d.	n.d.	n.d.	n.d.
	6	2.9E-01	1.8E-01	n.d.	3.2E-02	n.d.
	7	n.d.	n.d.	n.d.	n.d.	n.d.
	8	n.d.	n.d.	n.d.	n.d.	n.d.
	9	n.d.	1.2E-01	n.d.	6.0E-03	5.4E-03
**Prediabetes** (n = 4)	10	n.d.	1.3E-02	3.5E-03	n.d.	n.d.
	11	n.d.	5.3E-02	n.d.	n.d.	n.d.
	12	1.3E-01	1.2E-01	7.4E-03	7.4E-02	2.1E-03
	13	n.d.	1.8E-02	6.1E-03	0.0E+00	7.0E-03
**T2D** (n = 9)	14	1.6E-02	2.4E-02	2.7E-01	n.d.	n.d.
	15	n.d.	8.5E-02	n.d.	n.d.	n.d.
	16	n.d.	n.d.	n.d.	n.d.	n.d.
	17	3.6E-02	1.4E-02	n.d.	2.7E-01	n.d.
	18	n.d.	n.d.	n.d.	n.d.	n.d.
	19	n.d.	n.d.	n.d.	n.d.	n.d.
	20	n.d.	n.d.	n.d.	n.d.	n.d.
	21	2.8E-03	n.d.	n.d.	1.4E-01	n.d.
	22	n.d.	n.d.	n.d.	n.d.	n.d.

Ach, acetylcholine; n/a, not available; n.d., not detected; *, outlier excluded from correlation plot.
Values are shown in pmole/μg protein unless otherwise noted.
Tryptamine and L-DOPA are not included in this table since they were not detected in any of the measured samples.

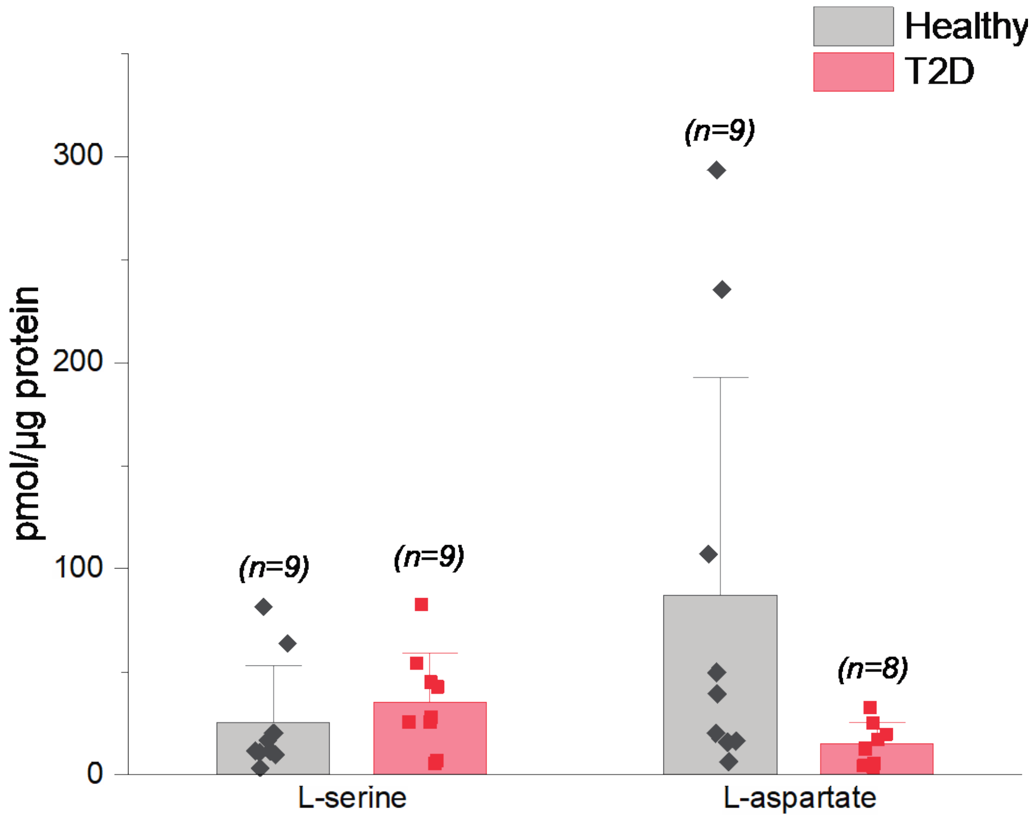

Figure S1. Levels of L-Ser and L-Asp in healthy versus type 2 diabetes (T2D)-affected human pancreatic islets. Values represent mean ± SD of 8-9 human islet samples.

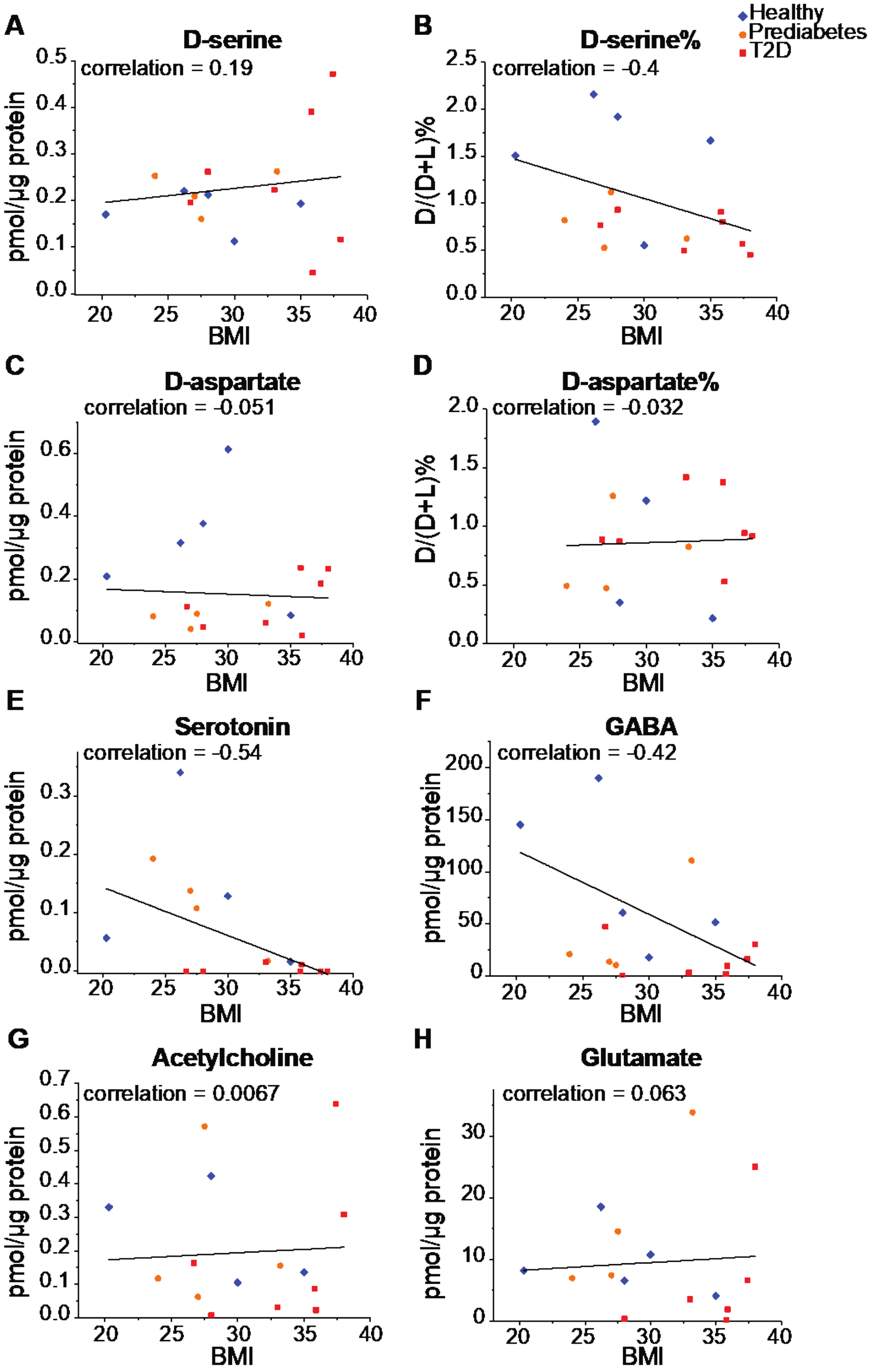

Figure S2. Scatter plots of BMI and each D-Ser, D-Asp, and classical neurotransmitters. The Pearson correlation coefficient is shown on each plot.

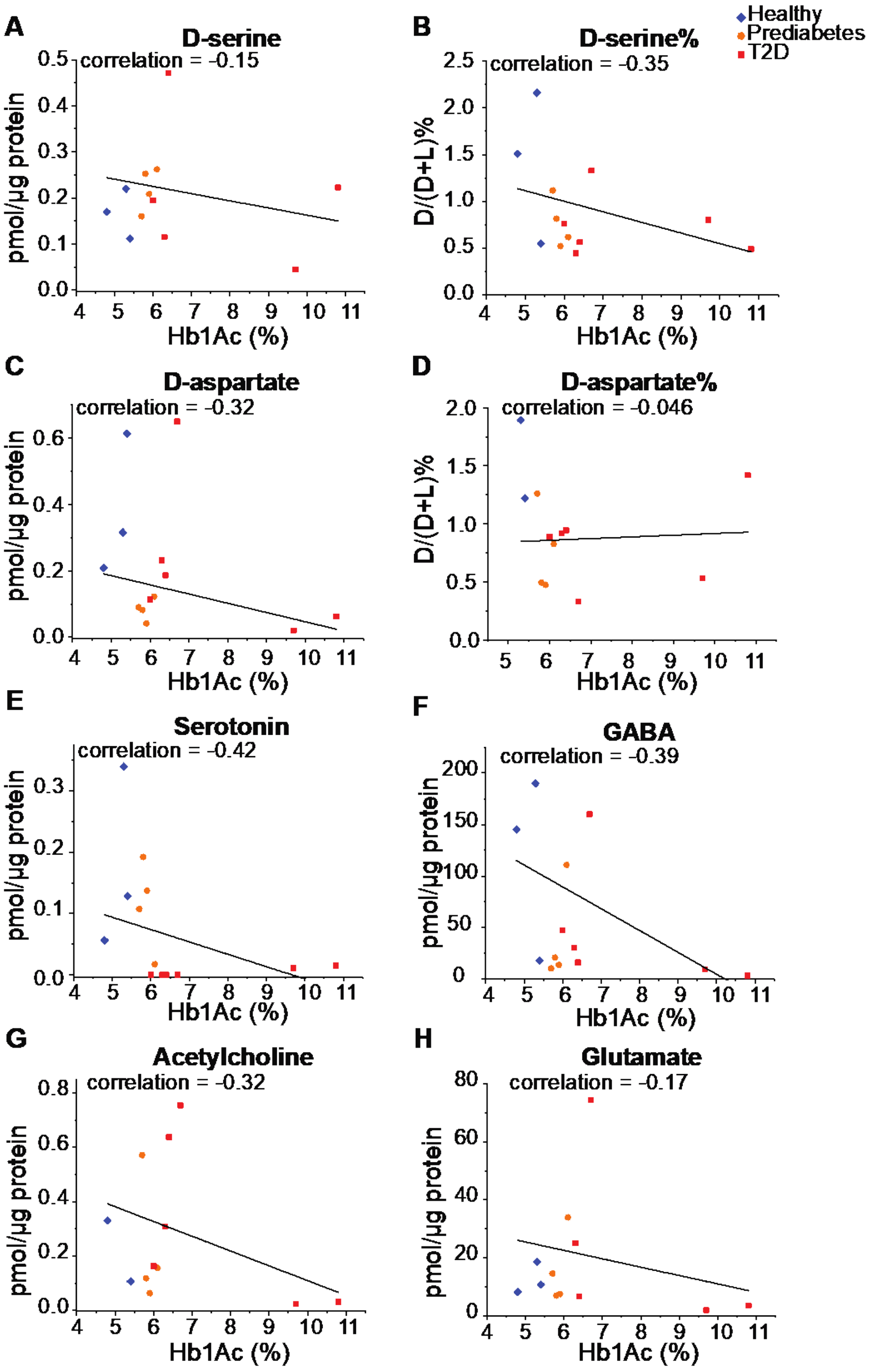

Figure S3. Scatter plots of HbA1c and each D-Ser, D-Asp, and classical neurotransmitters. The Pearson correlation coefficient is shown on each plot.
